# Lagged Effect of Parental Warmth on Child-to-Parent Violence through Moral Disengagement Strategies

**DOI:** 10.3390/children11050585

**Published:** 2024-05-11

**Authors:** Nazaret Bautista-Aranda, Lourdes Contreras, M. Carmen Cano-Lozano

**Affiliations:** Department of Psychology, University of Jaén, 23071 Jaén, Spain; lmcontre@ujaen.es (L.C.); mccano@ujaen.es (M.C.C.-L.)

**Keywords:** child-to-parent violence, moral disengagement, parental warmth, adolescents

## Abstract

Empirical evidence supports the simultaneous relationship between parental warmth and child-to-parent violence (CPV). However, no studies analyze the lagged effects of perceived parental warmth and the potential impact of cognitive mechanisms legitimizing immoral behavior on this relationship. This study aimed to examine the mediating role of moral disengagement strategies (reconstruction of immoral behavior, obscuring personal responsibility, misrepresenting injurious consequences, and blaming the victim) in the relationship between the perceived paternal and maternal warmth dimensions (warmth-communication and criticism-rejection) during childhood and CPV towards the father and mother. The sample included 2122 Spanish adolescents (57.7% female) aged 13 to 18 years. The Child-to-Parent Violence Questionnaire (CPV-Q), the Mechanisms of Moral Disengagement Scale (MMDS-S), and the Warmth Scale were used as assessment instruments. The results indicate that paternal and maternal warmth-communication is negatively associated with CPV, whereas paternal and maternal criticism-rejection and moral disengagement strategies are positively related to CPV. The mediation models show that the reconstruction of immoral behavior plays a crucial mediation role in the relationship between paternal and maternal warmth-communication and CPV as well as in the relationship between maternal criticism-rejection and CPV. The results emphasize the necessity of early prevention programs for parents promoting positive parenting practices, including parental warmth, to foster children’s adaptive socio-cognitive development. In addition, addressing moral disengagement in adolescents could help prevent or stop a pattern of violent behavior toward parents.

## 1. Introduction

In the violence perpetrated by children against their parents (child-to-parent violence, CPV), various forms of violent behaviors are manifested, such as physical, psychological, and economic violence, used to obtain power and control [1] against their parents or those who take their place [2]. Perpetrators engage in conscious, intentional, and repeated violent acts, excluding those resulting from psychological alterations or under a state of diminished consciousness [3]. This phenomenon constitutes a manifestation of violence within the family context, which has attracted increasing attention from the scientific community due to the alarming number of CPV complaints registered in the last decade. The General Prosecutor’s Office reported that 4332 judicial proceedings were initiated in Spain in 2022 [4]. However, these official data should be analyzed cautiously due to a high level of concealment on the part of affected parents, who are frequently reluctant to take the step of denouncing their children. Several studies with community samples of adolescents have shown a high presence of CPV cases in different countries (e.g., Ref. [5] in Germany; Ref. [6] in Spain; Ref. [7] in Mexico; Ref. [8] in Chile; and [9] in the United States), which allows us to have an approximation of the magnitude of the phenomenon in the general population. Specifically, it has been estimated, according to the reiterated criterion of aggressions, that the prevalence of physical violence is around 1.2% and 6.1%, psychological violence between 3.5% and 26.8%, and economic violence between 11.1% and 12.9%. In short, these figures highlight the seriousness of the phenomenon and emphasize the need to address this problem effectively.

CPV has a multicausal origin, influenced by various interacting variables at the individual, family, and social levels [10]. The family, being the first and most important socialization context for the child, significantly shapes subsequent aggressive behaviors. Therefore, numerous investigations have paid particular attention to the family dynamics in relation to CPV (e.g., Refs. [11,12,13]). One extensively explored aspect in this context is parenting styles, which Maccoby and Martin [14] recategorized as democratic, authoritarian, and permissive. These styles are classified by two key dimensions: (a) parental warmth/responsiveness and (b) parental control/demandingness, resulting in four parenting styles: authoritative, authoritarian, permissive-indulgent, and permissive-neglectful. Regarding the relationship between parenting style and CPV, research has consistently associated CPV with the authoritarian style [15,16,17]. In addition, the permissive styles have also been linked to CPV [16,18,19,20], particularly when CPV is motivated by instrumental reasons [21]. However, some studies have examined permissive-indulgent and permissive-neglectful separately, showing that Spanish adolescents who perpetrate aggression against their parents come to a greater extent from family contexts with a permissive-negligent style rather than from households with a permissive-indulgent style [15,17].

The complex relationship between parenting styles and CPV has led researchers to narrow their focus to specific parental dimensions or practices [10]. An essential variable of parenting style is parental warmth, which refers to responsiveness, affection, acceptance, and support that parents manifest for the needs of their children [14,22]. In contrast, parental rejection represents the absence of or significant withdrawal of these feelings and behaviors [23]. The Interpersonal Acceptance–Rejection Theory (IPARTheoty, [23,24,25]) substantiates that parental warmth and rejection have profound implications for an individual’s psychological adjustment, subsequent behavior, and relationships throughout life. This theory postulates that children are predisposed to develop a specific set of personality dispositions as a result of experiencing any combination of the following parental acceptance-rejection expressions: (1) cold and unaffectionate (the opposite of being warm and affectionate); (2) hostile and aggressive; (3) indifferent and neglecting; and (4) undifferentiated rejecting. Individuals have an emotional need to receive a positive response—support, affection, care, comfort, and attention—from people who are significant to them, such as parents in childhood. When this need is unmet due to experiences of rejection, individuals may develop psychological and behavioral responses of maladjustment, including hostility, aggression, emotional unresponsiveness, impaired self-esteem and self-adequacy, emotional instability, and negative worldview. Empirical evidence supports the probable causality of the long-term effects of perceived parental acceptance-rejection on psychological maladjustment and externalizing problems. Rothenberg et al. [26] conducted a seven-year longitudinal study involving a large sample of children aged 7 to 14 years from nine different countries. Their research demonstrated that all forms of paternal and maternal rejection predicted children’s internalizing and externalizing behaviors across all ages. Internalizing behaviors encompassed emotions and behaviors such as loneliness, self-consciousness, nervousness, sadness, and anxiety while externalizing behaviors encompassed acts such as lying, vandalism, bullying, disobedience, and physical violence.

In relation to CPV, previous studies have revealed that parental warmth and perceived emotional rejection seem to constitute core elements [16,27]. Specifically, the absence of parental warmth predicted verbal and physical CPV [18,28]. In the judicial context, Contreras and Cano-Lozano [29] identified that what differentiated juveniles accused of CPV offenses from other juvenile offenders and non-offenders was that CPV juveniles perceived less warmth and more criticism-rejection from parents than the other groups. Other research highlights the importance of maternal warmth. For instance, Ibabe et al. [16] found that CPV was associated with emotional rejection by the mother, but no significant association was observed with emotional rejection by the father. More recently, Zhang et al. [30] found that maternal emotional warmth was negatively associated with adolescent contempt and rebellion against their mother. Conversely, maternal rejection was positively associated with adolescent rebellion against their mother.

Several studies have analyzed how parental warmth and parental rejection are associated with CPV during the same temporal period [16,18,27,29]. However, a three-year longitudinal study with a large sample of Spanish adolescents revealed that the lack of parental warmth in the first year of the study correlated with CPV towards fathers and mothers over time, spanning the second and third years of the study follow-up [31]. These results could suggest that low parental warmth has a long-term effect on the development of CPV. To date, no research has ascertained the distant effects that parental warmth during childhood might have on CPV.

In short, the lack of parental warmth significantly impacts the development of violent behavior by adolescents toward their parents; however, as a risk factor, it does not explain by itself the CPV. Therefore, it would be interesting to analyze other variables implicated in this relationship. Recent research has highlighted the role of socio-cognitive variables [20,32,33]. Along these lines, Cano-Lozano et al. [11] examined the role of cognitive and emotional variables in the relationship between paternal and maternal warmth and CPV in a wide sample of adolescents from a community population. The results of this study indicated that parental warmth negatively correlated with hostile attribution and anger, while parental rejection correlated positively. These variables, in turn, positively correlated with CPV. Considering the significant impact of socio-cognitive factors on CPV and the limited research on this issue, it would be valuable to examine what other variables could be involved in the relationship between early parental warmth and CPV.

Moral disengagement is a socio-cognitive variable of particular relevance due to its connection with violent and antisocial behavior. Over the course of the socialization process, individuals acquire moral standards that guide their behavior, helping them to distinguish between what is socially correct or incorrect. Acting in line with internal standards provides satisfaction and avoids self-sanctions [34]. However, self-regulatory processes can be deactivated by different socio-cognitive mechanisms that promote the transgression of social norms and the appearance of immoral behaviors. The deactivation of this self-regulatory process is denominated moral disengagement, referring to the use of socio-cognitive mechanisms that allow the individual to justify and legitimize immoral behaviors and avoid negative self-evaluations and self-sanctions [34,35,36]. Bandura [34] identified eight mechanisms grouped into four strategies that represent the main points in the self-regulation process where internal moral control can be disengaged from immoral behavior. First, the reconstruction of immoral behavior operates to reinterpret prejudicial behavior by making it personally and socially acceptable via three specific mechanisms: moral justification, euphemistic labeling, and advantageous comparison. The second strategy refers to obscuring personal responsibility, which minimizes one’s responsibility for prejudicial behavior by two specific mechanisms: diffusion of responsibility and displacement of responsibility. The third strategy is misrepresenting injurious consequences, which includes the mechanism of distortion of consequences and operates to disregard or distort the harmful consequences of the transgressive behavior. The fourth and final strategy of moral disengagement, called blaming the victim, serves to modify perceptions of the victim via two specific mechanisms: dehumanization and attribution of blame. It should be noted that moral disengagement can manifest itself at various stages of life, even in the early years, although it is intensified and consolidated during adolescence when significant transformations in identity, independence, and social relations are experienced [35,37,38].

The empirical literature has revealed that moral disengagement is negatively related to prosocial behaviors [37,39,40] and positively related to violence in children and adolescents [37,41,42]. In particular, moral disengagement has been positively associated with various forms of violence during adolescence, such as bullying and cyberbullying [36,43] and also to dating violence [44,45]. More specifically, moral disengagement has been studied as a self-regulation process of behavior, according to contributions of the social learning theory [46]. Thus, from this approach, moral disengagement could act as a mediating variable. Some studies have analyzed the effect of an individual’s family environment, such as positive parenting [47], parental attachment [48], parent-adolescent conflict [49] or childhood maltreatment [50,51], on aggressive and delinquent behaviors through moral disengagement. In addition, the relationship between parental warmth and aggressive behavior has been mediated by moral disengagement, as suggested by some studies. For example, a longitudinal study with infants found that rejecting parenting showed a significant indirect effect on antisocial behavior through moral disengagement, whereas parental rejection was not directly related to antisocial behavior [52]. Recently, Zhang et al. [53] found that moral disengagement partially mediated the relationship between parental rejection and being cyber-aggressive, and, also, fully mediated the relationship between parental emotional warmth and cyber-aggressive behavior. Regarding CPV, to our knowledge, Bautista-Aranda et al. [54] conducted the first study to provide evidence that moral disengagement is a socio-cognitive mechanism associated with the violent behavior of adolescents toward their parents. Also, this study demonstrates that moral disengagement mediates the negative effect of exposure to family violence during childhood, both vicarious and direct victimization, on CPV. However, so far, there are no studies that analyze the effect of parental warmth during childhood on CPV through moral disengagement, so exploring this relationship would be interesting in this context.

**Figure 1:**
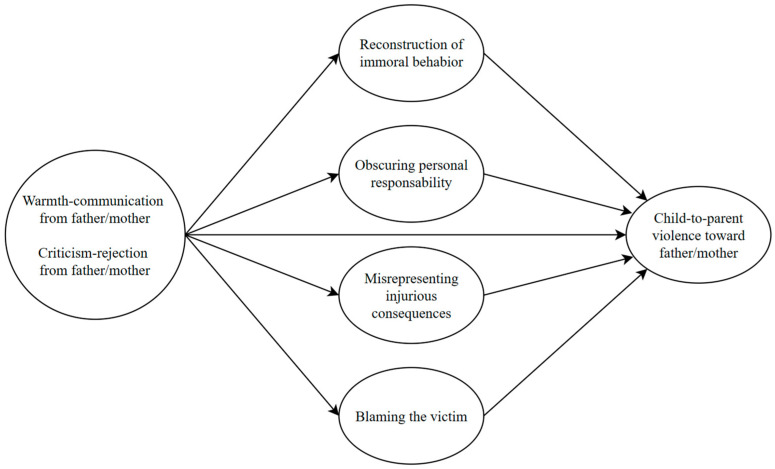
Conceptual mediational model for child-to-parent violence.

Based on the literature review, previous research has examined the individual impacts of parental warmth and rejection and moral disengagement on CPV [16,18,20,28,31,54]. However, there is a lack of studies adopting an integrated framework to understand the complex interrelationship between these factors. Based on the limitations identified in the literature, this research intends to clarify the relationship between the dimensions of perceived parental warmth during childhood separately (warmth-communication and criticism-rejection) and CPV through moral disengagement strategies (reconstruction of immoral behavior, concealment of personal responsibility, misrepresentation of harmful consequences, and victim blaming) within a comprehensive model (see conceptual mediational model, Figure 1). Specifically, the first objective was to examine the relationship between the perceived paternal and maternal warmth dimensions during childhood and CPV toward the father and mother. The second objective was to analyze the associations between moral disengagement strategies and CPV toward both father and mother. Lastly, the third objective was to explore whether different moral disengagement strategies mediate the relationship between dimensions of perceived paternal and maternal warmth during childhood and CPV toward both parents. Consequently, we proposed the following hypotheses.

**Hypothesis 1.**  *Perceived paternal and maternal warmth-communication during childhood will be negatively associated with CPV toward the father and the mother.*

**Hypothesis 2.**  *Perceived paternal and maternal criticism-rejection during childhood will be positively correlated to CPV against both the father and mother.*

**Hypothesis 3.**  *Moral disengagement strategies will be significantly and positively related to CPV toward both father and mother.*

**Hypothesis 4.**  *Moral disengagement strategies will mediate the relationship between perceived paternal and maternal warmth-communication during childhood and CPV toward the father and the mother.*

**Hypothesis 5.**  *Moral disengagement strategies will mediate the relationship between perceived paternal and maternal criticism-rejection during childhood and CPV against the father and mother.*

## 2. Materials and Methods

### 2.1. Participants

The sample comprised 2122 Spanish adolescents (57.7% female, 42.3% male) aged between 13 and 18 years (Mage = 14.9, SD = 1.3) from a community population. Participants were recruited from 25 high schools located in the provinces of Ciudad Real (60.1%), Córdoba (24.6%), Granada (9.8%), and Asturias (5.3%) (Spain) during the school years 2021/2022. Most of the sample (97.5%) was Spanish. Regarding the family structure of the participants, the majority of them reported that their parents lived together (83.9%), while 14.6% reported that their parents were divorced or separated. The socioeconomic levels were as follows: 9.9% high, 57.5% medium sufficient, and 4.6% low sufficient.

### 2.2. Instruments

The Child-to-Parent Violence Questionnaire (CPV-Q; Ref. [55]) consists of 14 parallel items (14 items for the father, *α* = 0.67, and 14 items for the mother, *α* = 0.70) that evaluate different acts of psychological (4 items), physical (3 items), and financial violence (3 items), as well as control and domain behaviors over parents (4 items). Adolescents were asked to indicate the frequency of perpetrating each behavior towards their parents during the last year using a 5-point scale ranging from never to very often (6 times or more).

The Warmth Scale, child’s version [56], is made up of 20 items, divided into two factors: warmth-communication (father: *α* = 0.91 and mother: *α* = 0.88) and criticism-rejection (father: *α* = 0.84 and mother: *α* = 0.78) by parents toward their children. Each factor consists of 10 items rated on a scale with a 5-point scale ranging from never to always. Adolescents were asked to report about perceived warmth from both fathers and mothers during childhood (before the age of 10 years).

The Mechanisms of Moral Disengagement Scale (MMDS; [37]. Spanish validation; MMDS-S; Ref. [57]) consists of 32 items referring to the eight mechanisms of moral disengagement and the corresponding four strategies: reconstruction of immoral behavior (includes moral justification, euphemistic labeling, and advantageous comparison), obscuring personal responsibility (includes diffusion of responsibility and displacement of responsibility), misrepresenting injurious consequences (includes distortion of consequences) and blaming the victim (includes dehumanization and attribution of blame). Each mechanism is composed of 4 items, each of which was answered with a 5-point scale ranging from fully disagree to fully agree. The Cronbach’s *α* was 0.76 for the reconstruction of immoral behavior, 0.60 for obscuring personal responsibility, 0.56 for misrepresenting injurious consequences, and 0.66 for blaming the victim.

### 2.3. Procedure

First, we obtained a favorable report from the Ethics Committee of the University of Jaén, Spain. Subsequently, we obtained authorizations from the Public Administration of Education and the directors of secondary education centers. Various secondary education centers were invited to participate and were given detailed information on the objectives of the research. The secondary centers that expressed their interest and availability to participate in the study provided informed consent on paper to both parents and children. For participants under 18 years of age, both the participants and their parents were required to give informed consent to participate in the study. Each assessment protocol the participant completed was assigned a unique identifying code. These codes were applied randomly and were not linked to personally identifiable information. Participation was voluntary, anonymous, and confidential, and no incentive was offered for participation. The investigators administered the questionnaires on paper in groups in the participant’s classrooms for approximately one hour.

### 2.4. Data Analysis

Descriptive analyses were conducted, including means and standard deviations, as well as Spearman correlations to determine the relationships between the study variables. In addition, the reliability of each of the instruments was evaluated by calculating Cronbach’s alpha coefficient. Data were analyzed using structural equation modeling using STATA v. 16 software. Two models were fitted: one to explore the relationship between perceived paternal and maternal warmth-communication during childhood and CPV through moral disengagement strategies and one to examine the association between perceived paternal and maternal parental criticism-rejection during childhood and CPV through moral disengagement strategies. Both models were replicated, on the one hand, for child-to-parent violence toward the father and, on the other hand, for child-to-parent violence toward the mother. We estimated the parameters using the maximum likelihood estimation method. The model fit was estimated using conventional indicators, such as root mean square error approximation (RMSEA), the Comparative Fit Index (CFI), the Tucker–Lewis Index (TLI), and standardized root mean square residual (SRMR). Following Hu and Bentler [58], a model is considered to fit adequately if the following reference values are reached or approached: a cutoff value close to or above 0.95 for CFI and TLI, a cutoff value close to or below 0.06 for RMSEA, and a cutoff value close to or below 0.08 for SRMR.

## 3. Results

Table 1 displays the means, standard deviations, and correlations between the variables examined in this study. CPV toward fathers and mothers was negatively and significantly related to paternal and maternal warmth-communication, while it was positively and significantly associated with paternal and maternal criticism-rejection. Likewise, CPV was positively and significantly correlated to all moral disengagement strategies (reconstruction of immoral behavior, obscuring personal responsibility, misrepresenting injurious consequences, and blaming the victim). The highest coefficients were found for the relationship between the reconstruction of immoral behavior and CPV towards both the father (*ρ* (2122) = 0.24, *p* < 0.001) and the mother (*ρ* (2122) = 0.24, *p* < 0.001).

**Figure 2:**
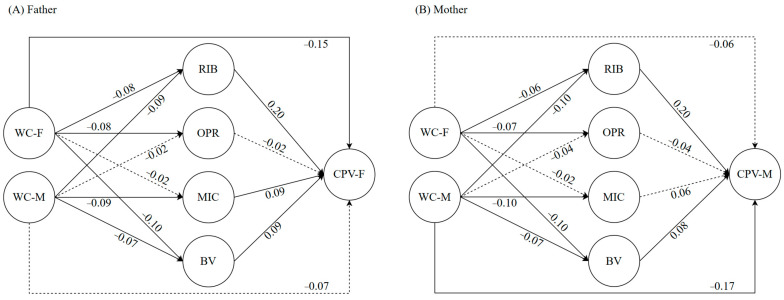
Results of mediational model 1 about the relationship between parental warmth-communication and child-to-parent violence. The circles represent the latent variables, and the arrows indicate the regression between variables, where the solid arrows indicate significant relationships (*p* < 0.05) and the dotted arrows represent non-significant relationships. The numbers indicate the standardized coefficient load of each variable in the model. WC = warmth-communication, RIB = reconstruction of immoral behavior, OPR = obscuring personal responsibility, MIC = misrepresenting injurious consequences, BV = blaming the victim, CPV = child-to-parent violence, F = father, and M = mother. The model for fathers is presented in (**panel A**), and the model for mothers is presented in (**panel B**).

The mediational model for the relationship between paternal and maternal warmth-communication and CPV through moral disengagement strategies is shown in Figure 2, which represents the results of the direct effects between the variables. This model was replicated for both CPV towards the father (Figure 2A) and CPV towards the mother (Figure 2B). The results analysis revealed a good fit for the model applied to fathers (*χ*^2^ (20) = 72.753, *p* < 0.001, *CFI_SB* = 0.977, *TLI_SB* = 0.949, *RMSEA_SB* = 0.036, *SRMR* = 0.028), accounting for 7.47% of the variance of child-to-father violence. For the mother’s model, the results also presented a good fit (*χ*^2^ (20) = 96.091, *p* < 0.001, *CFI_SB* = 0.968, *TLI_SB* = 0.930, *RMSEA_SB* = 0.042, *SRMR* = 0.029), explaining 7.95% of the variance for child-to-mother violence. Table 2 shows the effects in detail and the results of the indirect effects. It is observed that the reconstruction of immoral behavior significantly mediated both the relationship between paternal warmth-communication and CPV toward the father (*β* = −0.002, *SE* = 0.001, *p* < 0.05) and the relationship between maternal warmth-communication and CVP toward the father (*β* = −0.003, *SE* = 0.001, *p* < 0.05). These same results were found for CPV toward the mother (paternal warmth-communication: *β* = −0.002, *SE* = 0.001, *p* < 0.05; maternal warmth-communication: *β* = −0.004, *SE* = 0.001, *p* < 0.01). No significant mediation was observed with other moral disengagement strategies, namely obscuring personal responsibility, misrepresenting harmful consequences, and blaming the victim.

**Figure 3:**
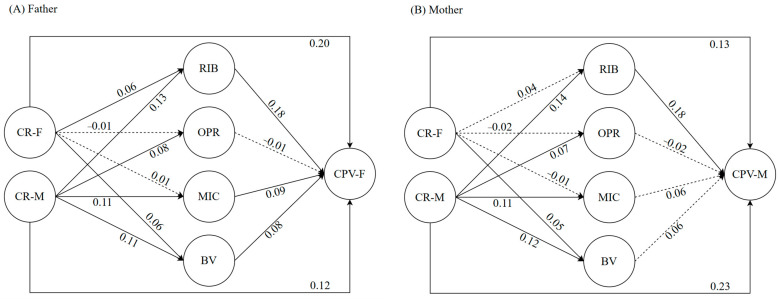
Results of mediational model 2 about the relationship between parental criticism-rejection and child-to-parent violence. The circles represent the latent variables, and the arrows indicate the regression between variables, where the solid arrows indicate significant relationships (*p* < 0.05) and the dotted arrows represent non-significant relationships. The numbers indicate the standardized coefficient load of each variable in the model. CR = criticism-rejection, RIB = reconstruction of immoral behavior, OPR = obscuring personal responsibility, MIC = misrepresenting injurious consequences, BV = blaming the victim, CPV = child-to-parent violence, F = father, and M = mother. The model for fathers is presented in (**panel A**), and the model for mothers is presented in (**panel B**).

Figure 3 shows the results of the mediational model for the relationship between paternal and maternal criticism-rejection and CPV mediated by moral disengagement strategies. Like the previous model, this model was also applied separately for the father (Figure 3A) and the mother (Figure 3B). The results showed an excellent model fit for both the father (*χ*^2^ (20) = 44.473, *p* < 0.005, *CFI_SB* = 0.989, *TLI_SB* = 0.976, *RMSEA_SB* = 0.024, *SRMR* = 0.020) and the mother (*χ*^2^ (20) = 71.063, *p* < 0.001, *CFI_SB* = 0.978, *TLI_SB* = 0.951, *RMSEA_SB* = 0.035, *SRMR* = 0.025). As for the structural part, the model explained 12.77% of the variance of child-to-father violence and 14.26% of the variance of child-to-mother violence. The indirect effects found in both models are shown in Table 3. It is identified that the reconstruction of immoral behavior mediated the effect of maternal criticism-rejection on CPV toward both father (*β* = 0.006, *SE* = 0.002, *p* < 0.01) and mother (*β* = 0.008, *SE* = 0.002, *p* < 0.01).

## 4. Discussion

The purpose of this research was to analyze whether moral disengagement strategies would mediate the relationship between the perceived parental warmth dimensions during childhood and CPV towards the father and mother. To address this objective, the first aim of this study was to examine the connection between perceived paternal and maternal warmth dimensions separately (warmth-communication and criticism-rejection) during childhood and CPV against both the father and mother. Our findings confirmed Hypothesis 1, showing that both paternal and maternal perceived warmth-communication during childhood was negatively correlated with CPV toward the father and the mother. These results are consistent with prior research linking CPV and low parental warmth [18,20,28,29,30,31]. In addition, we observed a significant association between perceived criticism-rejection from fathers and mothers during childhood and CPV toward both parents, which supported Hypothesis 2 and was in line with previous research indicating a positive relationship between parental rejection and CPV [16,29,30]. However, until now, few studies have delimited the time period during which parental warmth has been perceived, and it is difficult to determine whether it has an immediate or lagged effect on children’s aggressive behavior towards their parents. Our results are significant and go beyond previous research, demonstrating that both warmth-communication and criticism-rejection perceived from the father and mother during childhood (when children were younger than 10 years old) are associated with CPV toward the father and toward the mother. This suggests that the manner in which parents expressed warmth or rejection during childhood could influence adolescents’ behavior toward their parents at later stages, establishing a distant effect between early parental warmth and CPV. These results are in line with the IPAR Theory, which postulates that, when children experience rejection by their parents or other attachment figures, they are likely to develop psychological maladjustments over their lifetime, including internalizing and externalizing behaviors such as aggression [26]. In essence, negative early parenting experiences can have a lasting impact on individuals’ lives.

The second objective was to analyze the relationship between the four types of moral disengagement strategies (reconstruction of immoral behavior, obscuring personal responsibility, misrepresenting injurious consequences, and blaming the victim) and CPV. The results revealed that all moral disengagement strategies are positively and significantly related to violence toward both the father and the mother. This finding confirmed Hypothesis 2, and it is consistent with previous research showing that moral disengagement is positively associated with antisocial and transgressive behavior in adolescents [41,59], particularly, with child-to-father violence and child-to-mother violence [54]. Our results, congruent with Bandura’s theory [35,38], indicate that adolescents prone to employ different moral disengagement strategies are more likely to engage in violent behaviors toward their parents. These strategies allow adolescents to (1) transform their cognitive perception of violent behavior into good and socially acceptable behavior—reconstruction of immoral behavior; (2) minimize their responsibility for violent acts—obscuring personal responsibility; (3) distort the harmful consequences of their aggressive behaviors—misrepresenting injurious consequences; and (4) blame and devalue the victim/s —blaming the victim—in this case, their parents, making them unjustly responsible for their violent acts. Furthermore, the results show that the most intense correlations were between the reconstruction of immoral behavior and CPV, both in the cases of fathers and mothers. Similar data are reported in previous studies [60,61] that demonstrated that reconstruction of immoral behavior is the most effective psychological mechanism for disengagement of moral self-sanctions, compared to other strategies of moral disengagement. This is because cognitive reinterpretation not only allows individuals to eliminate self-sanctions but also to use self-approval to justify their destructive actions [34,35]. In other words, adolescents may engage in these harmful acts without experiencing personal distress and moral questioning, as they transform what was once considered morally unacceptable into becoming a source of motivation and positive self-valuation. Moreover, this dynamic could contribute to the persistence and escalation of violent behaviors perpetrated by adolescents against their parents.

The third aim was to explore the relationship between the dimensions of perceived paternal and maternal warmth (warmth-communication and criticism-rejection) and CPV through different moral disengagement strategies. The results found in previous research show that moral disengagement mediates the relationship between an individual’s family environment and aggressive and delinquent behaviors [47,48,49,50,51], including violent behaviors perpetrated against parents [54], and we expected that different moral disengagement strategies would mediate the effect of dimensions of perceived parental warmth during childhood on CPV. Our study showed that the dimensions of perceived parental warmth by the father and the mother have different indirect effects on CPV depending on whether the violence was directed toward the father or toward the mother.

On the one hand, the results partially confirmed Hypothesis 4; moral disengagement strategies will mediate the relationship between perceived paternal and maternal warmth-communication during childhood and CPV toward both parents. In the case of violence toward the father, the reconstruction of immoral behavior partially mediated the relationship between paternal warmth-communication and CPV and fully the relationship between maternal warmth-communication and CPV. Similarly, in the case of violence toward the mother, it was found that the reconstruction of immoral behavior totally mediated the relationship between paternal warmth-communication and CPV and partially the relationship between maternal warmth-communication and CPV. These findings indicate that violence toward a parent is directly related to perceived warmth during childhood by the same parent (i.e., between the warmth from the father and CPV toward the father and between the warmth from the mother and CPV toward the mother), and also indirectly through the reconstruction of immoral behavior. This means that, when adolescents have perceived their parent as unwarm and uncommunicative during their childhood, they are more prone to develop violent behaviors toward this same parent. This relationship is influenced by how adolescents reinterpret their behavior using the strategy of reconstructing immoral behavior. In contrast, perceived warmth by the non-victim parent is related to CPV through the reconstruction of immoral behavior. That is, maternal warmth is associated with CPV toward the father through the reconstruction of immoral behavior, while paternal warmth operates in the same way concerning CPV toward the mother. In these cases, the effect of parental warmth on CPV is only explained in the presence of the reconstruction of immoral behavior. Therefore, this result highlights the pivotal role of the reconstruction of immoral behavior strategy in understanding this relationship.

On the other hand, we expected that different moral disengagement strategies mediate the relationship between perceived paternal and maternal criticism-rejection during childhood and CPV toward both parents (Hypothesis 5). Our findings partially verified this supposition. To be more specific, it was obtained that reconstruction of immoral behavior partially mediated the association between perceived maternal criticism-rejection during childhood and CPV both towards the father and the mother. This finding suggests that, when adolescents have perceived that their mothers have criticized or rejected them during childhood, they may be more likely to engage in violent behavior toward both the father and the mother. This relationship is influenced in part by how they redefine their violent behavior; that is, how they justify and legitimize their behavior as becoming valid and acceptable through moral disengagement strategies such as reconstructing the violent behavior. In contrast, the results were not as expected with respect to the association between perceived paternal criticism-rejection during childhood and CPV toward the father and the mother through the four strategies of moral disengagement. In other words, the influence of paternal criticism-rejection on CPV was direct in both the father and mother models and was not mediated by moral disengagement strategies. This finding indicates that adolescents who have perceived their fathers as critical may show a more immediate and direct response in the form of violence against them, without the need to rationalize or justify their violent behaviors.

## 5. Conclusions

The present study has not only shown that all four types of moral disengagement strategies are associated with CPV but also that specifically the strategy of reconstructing immoral behavior has a crucial role as a mediator in the relationship between the dimensions of parental warmth and CPV. These results are consistent with previous studies indicating that moral disengagement mediated the link between family factors and CPV [54]. In particular, we observe that the relationship between perceived parental warmth-communication/criticism-rejection during childhood and CPV is better explained when the reconstruction of immoral behaviors is present. This applies in (a) the relationship between paternal warmth-communication and CPV toward the father, (b) the relationship between maternal warmth-communication and CPV toward the mother, and (c) the relationship between maternal criticism-rejection and CPV toward the father and toward the mother. In contrast, the relationship between paternal warmth-communication and CPV toward the mother, and maternal warmth-communication and CPV toward the father were only explained in the presence of the strategy of reconstructing immoral behaviors. Nevertheless, other moral disengagement strategies, such as reinterpreting the consequences of their violent actions, minimizing their responsibility, or blaming the victims did not play a significant role in these associations. In general, moral disengagement allows individuals to reinterpret their harmful actions, deactivate self-sanctions to make their actions appear less harmful or even harmless, and clear the way for perpetrating transgressions [37,46,59]. Our results might indicate that, in cases of CPV, adolescents who have experienced low paternal and maternal warmth and maternal rejection during childhood are more likely to employ moral disengagement strategies when committing violence toward their fathers and mothers. In other words, through the cognitive reinterpretation of their violent acts, they justify and legitimize their actions, making them acceptable and, thus, avoiding guilt, distress, or remorse. This, in turn, promotes the occurrence of CPV.

It is important to note some limitations that should be considered when interpreting the results of this study. Firstly, the participants in this research are adolescents from four Spanish provinces, which affects the generalizability of the findings to other geographical areas, both nationally and internationally. To address this limitation, we suggest increasing the geographic diversity of the sample to ensure the representativeness of the results. Secondly, the results are based exclusively on self-reports by the adolescents themselves and, therefore, they refer to the perception they have of their parents. To enhance understanding, future research should incorporate the perspectives of parents and other family members, enabling a holistic analysis of family interactions and adolescent behavior. Lastly, this is a cross-sectional study, which means that it is not possible to establish causal relationships between the investigated variables. Therefore, longitudinal studies are suggested to explore the causal relationships between the investigated variables over time. This methodological approach would offer a more comprehensive understanding of the CPV phenomenon and clarify the direction of causal influences among the variables studied. In this sense, it highlights the need to conduct additional research that considers the family as an interconnected system, where the actions and relationships of each member impact the whole. In this sense, the family would be examined from a systemic perspective [62,63].

The findings obtained in this research carry several implications worth mentioning. This study contributes to our understanding of the cognitive mechanisms involved in behavioral self-regulation, linking perceived parental warmth in childhood to the manifestation of violent behaviors toward parents in adolescence. Furthermore, this study has provided evidence by exploring the relationship between different moral disengagement strategies and CPV, an aspect that has been scarcely explored. Therefore, these results should be considered when designing prevention and intervention. On the one hand, the findings support the idea of implementing early prevention programs aimed at parents, focusing on promoting positive parenting practices, such as the expression of warmth, support, and affection toward children. This approach aims to foster an adaptive socio-cognitive development of children since childhood, acting as a crucial preventive measure against the development of aggressive behaviors towards their parents in the future. On the other hand, for cases where CPV is already present, intervention programs should also address moral disengagement. This involves modifying beliefs and attitudes that approve or justify violence as a means of conflict resolution. The goal is to dismantle the cognitive bases that support CPV and, instead, promote healthy family relationships and prevent the emergence of violent behaviors toward parents.

## Figures and Tables

**Table 1:** Means, standard deviations, and Spearman correlations between study variables.

	*M*	*SD*	1	2	3	4	5	6	7	8	9	10
1. WC-F	28.75	9.39	1									
2. WC-M	32.49	7.29	0.68 ***	1								
3. CR-F	5.46	5.87	−0.48 ***	−0.36 ***	1							
4. CR-M	5.16	5.19	−0.34 ***	−0.47 ***	0.78 ***	1						
5. RIB	22.79	6.71	−0.17 ***	−0.17 ***	0.18 ***	0.20 ***	1					
6. OPR	20.51	5.51	−0.10 ***	−0.09 ***	0.03	0.07 **	0.43 ***	1				
7. MIC	7.63	2.77	−0.13 ***	−0.16 ***	0.12 ***	0.14 ***	0.56 ***	0.38 ***	1			
8. BV	15.62	4.99	−0.17 ***	−0.17 ***	017 ***	0.17 ***	0.60 ***	0.42 ***	0.45 ***	1		
9. CPV-F	5.01	4.17	−0.16 ***	−0.14 ***	0.30 ***	0.27 ***	0.24 ***	0.14 ***	0.18 ***	0.21 ***	1	
10. CPV-M	5.65	4.58	−0.18 ***	−0.18 ***	0.26 ***	0.31 ***	0.24 ***	0.13 ***	0.16 ***	0.20 ***	0.84 ***	1

Note. ** *p* < 0.01, *** *p* < 0.001. WC = warmth-communication, CR = criticism-rejection, RIB = reconstruction of immoral behavior, OPR = obscuring personal responsibility, MIC = misrepresenting injurious consequences, BV = blaming the victim, CPV = child-to-parent violence, F = father, and M = mother.

**Table 2:** Mediation effects of parental warmth-communication on child-to-parent violence.

Path	Coeff.	Std. Err.	*z*	*p*	95% Conf. Interval	
					LLCI	ULCI
Child-to-father violence						
WC-F → RIB → CPV-F	−0.0023	0.0010	−2.36	0.018	−0.0042	−0.0004
WC-F → OPR → CPV-F	0.0003	0.0004	0.68	0.498	−0.0005	0.0010
WC-F → MIC → CPV-F	−0.0003	0.0004	−0.79	0.432	−0.0011	0.0005
WC-F → BV → CPV-F	−0.0014	0.0007	−1.88	0.060	−0.0028	0.0001
WC-M → RIB → CPV-F	−0.0035	0.0014	−2.51	0.012	−0.0062	−0.0008
WC-M → OPR → CPV-F	0.0001	0.0002	0.54	0.587	−0.0003	0.0005
WC-M → MIC → CPV-F	−0.0016	0.0008	−1.88	0.060	−0.0032	0.0001
WC-M → BV → CPV-F	−0.0012	0.0007	−1.58	0.113	−0.0026	0.0003
Child-to-mother violence						
WC-F → RIB → CPV-M	−0.0021	0.0010	−2.03	0.042	−0.0041	−0.0001
WC-F → OPR → CPV-M	0.0004	0.0004	1.14	0.256	−0.0003	0.0011
WC-F → MIC → CPV-M	−0.0002	0.0003	−0.56	0.573	−0.0008	0.0004
WC-F → BV → CPV-M	−0.0013	0.0007	−1.85	0.064	−0.0027	0.0001
WC-M → RIB → CPV-M	−0.0047	0.0016	−2.82	0.005	−0.0079	−0.0014
WC-M → OPR → CPV-M	0.00031	0.0003	0.95	0.345	−0.0003	0.0010
WC-M → MIC → CPV-M	−0.0013	0.0009	−1.51	0.131	−0.0030	0.0004
WC-M → BV → CPV-M	−0.0012	0.0007	−1.62	0.105	−0.0027	0.0001

Note. WC = warmth-communication, RIB = reconstruction of immoral behavior, OPR = obscuring personal responsibility, MIC = misrepresenting injurious consequences, BV = blaming the victim, CPV = child-to-parent violence, F = father, and M = mother.

**Table 3:** Mediation effects of parental criticism-rejection on child-to-parent violence.

Path	Coeff.	Std. Err.	*z*	*p*	95% Conf. Interval	
					LLCI	ULCI
Child-to-father violence						
CR-F → RIB → CPV-F	0.0021	0.0014	1.79	0.074	−0.0003	0.0054
CR-F → OPR → CPV-F	0.0001	0.0001	0.12	0.902	−0.0002	0.0002
CR-F → MIC → CPV-F	0.0001	0.0007	0.15	0.879	−0.0012	0.0010
CR-F → BV → CPV-F	0.0011	0.0008	1.45	0.148	−0.0004	0.0027
CR-M → RIB → CPV-F	0.0060	0.0021	2.79	0.005	0.0018	0.0102
CR-M → OPR → CPV-F	−0.0001	0.0006	−0.13	0.895	−0.0012	0.0011
CR-M → MIC → CPV-F	0.0025	0.0014	1.86	0.062	−0.0001	0.0052
CR-M → BV → CPV-F	0.0023	0.0013	1.78	0.075	−0.0002	0.0049
Child-to-mother violence						
CR-F → RIB → CPV-M	0.0018	0.0014	1.29	0.196	−0.0009	0.0046
CR-F → OPR → CPV-M	0.0001	0.0002	0.50	0.620	−0.0003	0.0005
CR-F → MIC → CPV-M	−0.0001	0.0005	−0.11	0.910	−0.0010	0.0009
CR-F → BV → CPV-M	0.0009	0.0007	1.33	0.182	−0.0004	0.0022
CR-M → RIB → CPV-M	0.0081	0.0026	3.17	0.002	0.0031	0.0131
CR-M → OPR → CPV-M	−0.0005	0.0007	−0.70	0.487	−0.0018	0.0009
CR-M → MIC → CPV-M	0.0022	0.0014	1.55	0.120	−0.0006	0.0050
CR-M → BV → CPV-M	0.0023	0.0014	1.67	0.094	−0.0004	0.0051

Note. CR = criticism-rejection, RIB = reconstruction of immoral behavior, OPR = obscuring personal responsibility, MIC = misrepresenting injurious consequences, BV = blaming the victim, CPV = child-to-parent violence, F = father, and M = mother.

## Data Availability

The data presented in this study are available on request from the corresponding author due to confidentiality reasons.
